# Effects of Standardized Brazilian Green Propolis Extract (EPP-AF®) on Inflammation in Haemodialysis Patients: A Clinical Trial

**DOI:** 10.1155/2022/1035475

**Published:** 2022-11-22

**Authors:** Marcelo Augusto Duarte Silveira, Hayna Malta-Santos, Jéssica Rebouças-Silva, Flávio Teles, Erica Batista dos Santos Galvão, Sergio Pinto de Souza, Fábio Ricardo Dantas Dutra, Marcel Miranda Dantas Gomes, Maurício Brito Teixeira, Luis Filipe Miranda Rebelo da Conceição, Carolina Sa Nascimento, Carolina Kymie Vasques Nonaka, Rodrigo Silva Cezar, Paulo Benigno Pena Batista, Andresa Aparecida Berretta, Valeria M. Borges, Rogerio da Hora Passos

**Affiliations:** ^1^D'Or Institute for Research and Education (IDOR), Hospital São Rafael, Nephrology Department, Avenida São Rafael 2152, São Marcos, Salvador, BA 41253190, Brazil; ^2^UNIME Medical School, Fazenda Pitangueira, Lauro de Freitas, Bahia 42700000, Brazil; ^3^Gonçalo Moniz Institute, Oswaldo Cruz Foundation (FIOCRUZ), Rua Waldemar Falcão 121, Candeal, Salvador, BA 40296710, Brazil; ^4^School of Medicine, Federal University of Bahia, Rua Augusto Viana s/n, Canela, Salvador, BA 40110909, Brazil; ^5^School of Medicine, Federal University of Alagoas, Av. Lourival de Melo Mota S/N, Tabuleiro do Martins 57072900, Maceió, Alagoas, Brazil; ^6^Escola Bahiana de Medicina e Saúde Pública-EBMSP, Av. Dom João VI 275, Brotas, Salvador, BA 40290000, Brazil; ^7^Universidade do Estado da Bahia (UNEB), Rua Silveira Martin 2555, Cabula, Salvador, BA 41150000, Brazil; ^8^Research, Development and Innovation Department, Apis Flora Indl. Coml. Ltd, Rua Triunfo 945, Subse-tor Sul 3, Ribeirão Preto, SP 14020670, Brazil

## Abstract

**Background:**

Patients on haemodialysis (HD) present a significant inflammatory status, which has a pronounced negative impact on their outcomes. Propolis is a natural resin with anti-inflammatory and immunomodulatory properties. We assessed the safety and impact of a standardized Brazilian green propolis extract (EPP-AF®) on the inflammatory status in patients under conventional HD.

**Methods:**

Patients were assigned to receive 200 mg/day of EPP-AF® for 4 weeks followed by 4 weeks without the drug, and changes in plasma levels of interleukins (ILs), interferon gamma (IFN-*γ*), tumour necrosis factor-alpha (TNF-*α*), and high-sensitivityc-reactive protein (HsCRP) were measured. A heatmap was used to illustrate trends in data variation.

**Results:**

In total, 37 patients were included in the final analysis. Patients presented an exacerbated inflammatory state at baseline. During EPP-AF® use, there was a significant reduction in IFN-*γ* (*p*=0.005), IL-13 (*p*=0.04 2), IL-17 (*p*=0.039), IL-1ra (*p*=0.008), IL-8 (*p*=0.009), and TNF-*α* (*p*  <  0.001) levels compared to baseline, and significant changes were found in Hs-CRP levels. The heatmap demonstrated a pattern of pronounced proinflammatory status at baseline, especially in patients with primary glomerulopathies, and a clear reduction in this pattern during the use of EPP-AF®. There was a tendency to maintain this reduction after suspension of EPP-AF®. No significant side effects were observed.

**Conclusion:**

Patients under haemodialysis presented a pronounced inflammatory status, and EPP-AF® was demonstrated to be safe and associated with a significant and maintained reduction in proinflammatory cytokines in this population. This trial is registered with Clinicaltrials.gov NCT04072341.

## 1. Introduction

In recent decades, chronic kidney disease (CKD) has gained a pandemic status [1]. Despite advances in its management, the mortality of CKD patients, especially on haemodialysis, remains high, mainly due to cardiovascular (CV) events [2, 3]. In addition to traditional CV risk factors (dyslipidaemia, sedentary lifestyle, obesity, and smoking), haemodialysis patients are subject to nontraditional factors (uraemic toxins, hypervolemia, bone disease, oxidative stress, anaemia, and malnutrition/inflammation syndrome), all of which are associated with an exacerbated and chronic inflammatory state, which induces unfavourable outcomes [2–5].

Recently, inflammation as a predictor of worse prognosis in haemodialysis patients has been recognized [2, 4]. Inflammatory markers, such as high-sensitivityC-reactive protein (hs-CRP), are higher in dialysis patients than in the general population, which may be a result of increased cytokines, such as interleukin-1 beta (IL-1*β*), tumour necrosis factor-alpha (TNF-*α*), and IL-6 [5, 6].

Due to the multifactorial nature of inflammation in CKD, it is unlikely that a single strategy could halt the entire process. In this sense, the following two therapeutic rationales are important: strategies aimed at the removal of cytokines, such as the use of dialysis techniques using diffusive and convective clearance (haemodiafiltration, HDF); new pharmacological therapies that act in the reduction or modulation of proinflammatory molecules [2]. HDF potentially removes proinflammatory cytokines from the circulation [2, 4, 7]. However, despite convective methods, the inflammatory status persists, and new therapies that modulate the production of inflammatory mediators are needed.

Propolis is a natural resin produced by bees from different parts of plants [8]. Propolis has numerous properties of interest in medicine, including antioxidant, anti-inflammatory, and immunomodulatory properties [8–13]. In experimental models and in humans with CKD, we recently demonstrated that the use of propolis reduces renal inflammatory processes and decreases proteinuria, which is considered a classic marker of renal function loss and cardiovascular risk [8–14].

However, it remains unknown whether propolis modulates the systemic inflammatory process, which cytokines are modulated by propolis and its safety in haemodialysis patients. Therefore, we designed the present study to assess safety and impact of a standardized Brazilian green propolis extract (EPP-AF®) on systemic inflammation in adults on haemodialysis.

## 2. Materials and Methods

### 2.1. Study Design and Oversight

The clinical study was a prospective, open-label, proof-of-concept, single-centre study conducted from August to December, 2019, at São Rafael Hospital, Salvador, Bahia, in northeast Brazil. The study was approved by the local Ethics Committees (registration number 12849619.9.0000.0048) on June 11, 2019. The study was registered on the Clinicaltrials.gov platform (NCT04072341) on August 28, 2019. The present study was conducted in accordance with the principles of the Declaration of Helsinki and the Good Clinical Practice guidelines of the International Conference on Harmonization.

After the approval of written consent, patients were assigned to receive 4 weeks of Propomax® capsules produced with dehydrated standardized Brazilian green propolis extract (EPP-AF®) [10] at a dose of 200 mg/day (one 100 mg capsule, twice daily) followed by 4 weeks of period control without EPP-AF®. There was a wash-out week between periods.

A standardized Brazilian green propolis extract, which is composed mainly of a green propolis produced in southeast Brazil and processed with a specific extraction and drying process, was selected for use in this study due to its batch-to-batch reproducibility [10]. The dosage of 200 mg/day offered 10.6 mg of total flavonoids, such as quercetin (measured according to procedures previously described) [15], as well as 27 mg of total phenolics, such as gallic acid [16].

The dose of propolis was selected based on previously completed clinical studies with EPP-AF® and with the evaluation of safety of the product [8, 13, 14]. Patients were instructed to take the propolis capsules after the dialysis sessions on the days of treatment so that there would be no interference with their action because removal of the compound by dialysis is unknown. Blood collections were performed at baseline and after each period. Each patient was his or her own control.

All authors guarantee data integrity and fidelity to the study protocol. This study was the initiative of the principal investigator and correspondent.

### 2.2. Patient Selection and Haemodialysis Modality

Adults on classic haemodialysis treatment for at least 1 month were included. The following patients were excluded from this analysis: patients who missed dialysis sessions, pregnant women, patients with propolis allergy, patients using short-term catheters, patients with recent fistula thrombosis (less than 30 days), patients undergoing kidney transplantation or some surgical treatment, patients using immunosuppressants, patients diagnosed with cancer or rheumatic diseases, and patients with active infections or need for hospitalization in the period.

All patients were under the same haemodialysis treatment throughout the study as follows: classic haemodialysis mode; 03 (three) times a week; blood flow ranging from 300 to 350 ml/min; average dialysate flow of 500 ml/min; capillary FX 100 classix or FX 80 classix, Fresenius 4008S machine (Fresenius Medical Care AG, Bad Homburg, Germany); and adjusted time sufficient for single pool KT/V greater than or equal to 1.2.

### 2.3. Participant Monitoring

Patients were monitored to ensure safety, which is the major premise of the entire study. To assess the potential toxicity of the compound, an adverse effect questionnaire (time to onset, duration, type, and severity) with questions directed by systems was established, and the researchers alerted patients to be informed of the first sign of new clinical changes as well as about the suspension of the use of the product.

Participants were evaluated during haemodialysis sessions and were followed up by telephone in the interdialytic period. Laboratory data for safety analysis were also collected. Data analysis was performed by an external and impartial statistician with no patient involvement. Adverse events that could compromise patient safety or considered serious were reported to the local research Ethics Committee, and the study was discontinued in this situation.

### 2.4. Laboratory Tests and Cytokine Analysis

All biochemical tests were analysed at the Hospital São Rafael Central Laboratory, a certified laboratory that follows international standards. Blood for analysis in each study period was always collected before the dialysis procedure. Hs-CRP analysis was performed by immunoturbidimetric assay.

For the evaluation of ILs, the blood of the patients was centrifuged, and the plasma was frozen and stored at −80°C for further analysis. Plasma levels of IFN-*γ*, IL-1*β*, IL-1*α*, IL-1RA, IL-4, IL-6, IL-8, IL-10, IL-12p70, IL-13, IL-17, and TNF-*α* were measured in cryopreserved EDTA plasma samples using a commercially available MILLIPLEX kit based on Luminex xMAP technology (Merck, Darmstadt, Germany) according to the manufacturer's instructions. The heatmap was used to illustrate trends in data variation over the studied periods. This approach was used to summarize the large numbers of comparisons.

To evaluate the potential for liver, pancreatic, or muscle toxicity, analyses were performed before and after the use of EPP-AF with aspartate aminotransferase (AST), alanine aminotransferase (ALT), amylase, and creatine phosphokinase (CPK) techniques according to conventional laboratory techniques.

### 2.5. Outcomes

The primary endpoint was the assessment of inflammatory parameters using the interleukin and hs-CRP panel. The secondary outcome was the safety assessment of EPP-AF through an adverse effect questionnaire and laboratory tests.

### 2.6. Statistical Analysis

Continuous variables are expressed as the mean and standard deviation (SD) or as the mean and 95% confidence interval (95% CI). Categorical variables are expressed as absolute and relative frequencies. Student's *t* test or *χ*^2^ test were used for parametric data and nonparametric variables, respectively. We used intention-to-treat analyses for the primary and secondary endpoints.

Wilcoxon signed-rank exact tests were performed to compare the IL; in the case of paired ranks, Wilcoxon signed-rank tests were performed with continuity correction. Effect sizes were calculated using the following formula: *Z*/(√*N*) [17].

The McNemar–Bowker test was used to evaluate the proportions in paired samples for the analysis of hs-CRP and stratified by the cut-off points of <1 mg/L, between 1 and 3 mg/L and greater than 3 mg/L, which were based on a previous study that correlated these ranges with low, moderate, and high cardiovascular risk, respectively [18].

Data for each biomarker were log-transformed, and the *z* score was normalized to build heatmaps to illustrate the overall trends of data variation in the studied periods. Hierarchical cluster analyses (Ward's method) were used to group the biomarkers with similar distributions between clinical groups and time points. In such analyses, dendrograms represent Euclidean distance. The data processing and analyses were performed in JMP Pro (version 13.0.0), Graph Pad Prism (version 8), or R (version 4.1.2).

## 3. Results

### 3.1. Study Population

**Figure 1:**
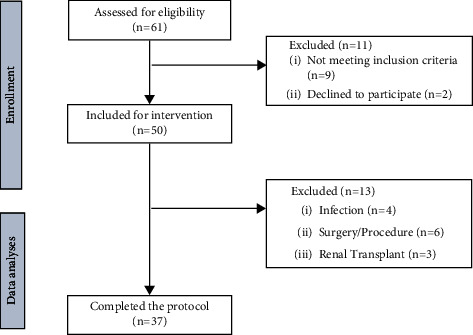
Flow diagram of study participants.

The baseline characteristics are shown in Table 1. We initially evaluated 61 patients and identified 50 eligible patients. Of these eligible patients, 13 patients were excluded due to infection, transplantation, or other surgical procedures, which could interfere with the assessment of inflammatory parameters. The flow diagram is shown in Figure 1.

The mean age ± standard deviation (SD) of the patients was 58.6 ± 15 years, and 22 (59%) of the patients were men. The main causes of ESRD were hypertension (37.8%), diabetes (24.3%), and glomerular disease (16.2%). The mean duration of haemodialysis (in months) was 24 months (interquartile range (IQR): 4–228), and the majority (70.3%) used arteriovenous fistula as the vascular access (Table 1).

### 3.2. Primary Outcomes

**Figure 2:**
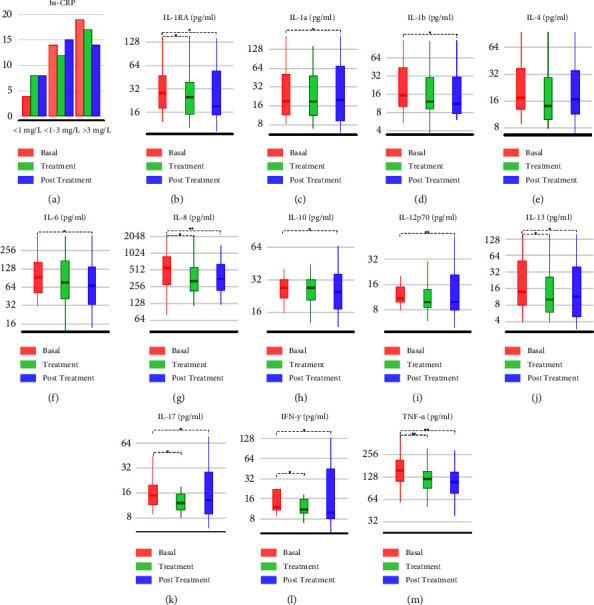
Inflammatory parameters of patients in the periods studied. ^∗^*p* <  0.05. ^∗∗^*p* <  0.001.

The distribution of individual values of inflammatory and immunological molecules is shown in Tables 2 and 3 and Figure 2.

Comparison of the interleukin levels of the baseline period versus the treatment period demonstrated decreased IFN-*γ* levels from 12 pg/ml (IQR, 11–22) to 11 pg/ml (IQR, 10–16) with EPP-AF treatment (*p* = 0.005 and an effect size of 0.47). IL-13 ranged from a baseline value of 14 pg/ml (IQR, 8–52) to 10 pg/ml (IQR, 6–26) with treatment (*p* = 0.042 and an effect size of 0.33). IL-17 varied from a baseline value of 15 pg/ml (IQR, 12–20) to 12 pg/ml (IQR, 10–16) with EPP-AF treatment (*p* = 0.039 and an effect size of 0.35). IL-1Ra ranged from a baseline value of 28 pg/ml (IQR, 18–47) to 25 pg/ml (IQR, 15–38) with EPP-AF treatment (*p* = 0.008 and an effect size of 0.44). IL-8 ranged from a baseline value of 556 pg/ml (IQR, 282–880) to 323 pg/ml (IQR, 215–566) with treatment (*p* = 0.009 and an effect size of 0.45). TNF- *α* ranged from a baseline value of 155 pg/ml (IQR, 112–215) to 120 pg/ml (IQR, 89–152) with EPP-AF treatment (*p* <0.001 and an effect size of 0.77). Regarding hs-CRP, the number of patients with low baseline cardiovascular risk (level <1 mg/L) was 11% and increased to 22% after using EPP-AF (*p* = 0.3). Other markers showed no significant differences between the baseline and after using EPP-AF (Tables 2 and 3).

**Figure 3:**
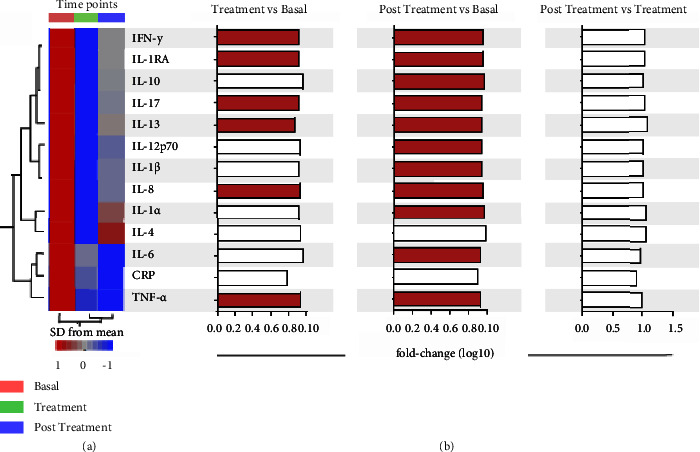
Heatmap demonstrating the inflammation profile of patients in the measured periods. The heatmap (a) shows data for the mean plasma concentration of each indicated marker per time point, and the data were log-transformed and *Z* score normalized. This approach was used to illustrate trends in data variation. A hierarchical cluster analysis (Ward's method with 100X bootstrap) was used to group the biomarkers with similar distributions between the time points. Dendrograms represent Euclidean distance. The red and blue colours demonstrate a higher and lower inflammatory pattern, respectively. The fold differences between the indicated means were calculated, and log10 values were plotted (b). Differences between each time point were examined using the Wilcoxon matched paired test. Red bars indicate mediators that were significantly different between the groups.

Heatmap analysis demonstrated a pronounced degree of inflammation at baseline and a clear reduction in inflammation after EPP-AF® treatment with a tendency to maintain this reduction in the period after discontinuation of medication (posttreatment) (Figure 3(a)). In addition, we also calculated fold differences in the concentration values of the cytokines between each time point as shown in Figure 3(b).

Comparison of baseline interleukin levels versus no treatment period (posttreatment; *t*3) demonstrated that there was maintenance of the reduction in the level of the following markers: IFN-*γ* from 12 pg/ml (IQR, 11–22) to 10 pg/ml (IQR, 8–46) (*p* = 0.003 and an effect size of 0.55); IL-10 ranged from a baseline value of 27 pg/ml (IQR, 22–32) to 25 pg/ml (IQR, 17–36) (*p* = 0.03 and an effect size of 0.39); IL-12p70 ranged from a baseline value of 11 pg/ml (IQR, 10–15) to 10 pg/ml (IQR, 8–21) (*p* < 0.001 and an effect size of 0.62); IL-13 ranged from a baseline value of 14 pg/ml (IQR, 8–52) to 12 pg/ml (IQR, 5–40) (*p* = 0.012 and an effect size of 0.45); IL-17 ranged from a baseline value of 15 pg/ml (IQR, 12–20) to 13 pg/ml (IQR, 9–28) (*p* = 0.003 and an effect size of 0.55); IL-1Ra ranged from a baseline value of 28 pg/ml (IQR, 18–47) to 19 pg/ml (IQR, 14–55) (*p* = 0.016 and an effect size of 0.43); IL-1*α* ranged from a baseline value of 19 pg/ml (IQR, 12–51) to 20 pg/ml (IQR, 9–71) (*p* = 0.033 and an effect size of 0.38); IL-1b ranged from a baseline value of 15 pg/ml (IQR, 10–44) to 11 pg/ml (IQR, 8–30) (*p* = 0.002 and an effect size of 0.56); IL-6 ranged from a baseline value of 93 pg/ml (IQR, 52–164) to 68 pg/ml (IQR, 33–137) (*p* = 0.003 and an effect size of 0.52); IL-8 ranged from a baseline value of 556 pg/ml (IQR, 282–880) to 358 pg/ml (IQR, 222–643) (*p* < 0.001 and an effect size of 0.70); TNF-*α* ranged from a baseline value of 155 pg/ml (IQR, 112–215) to 109 pg/ml (IQR, 76–146) (*p* < 0.001 and an effect size of 0.85). The other markers showed no significant differences between baseline and *t*3 (Table 2).

**Figure 4:**
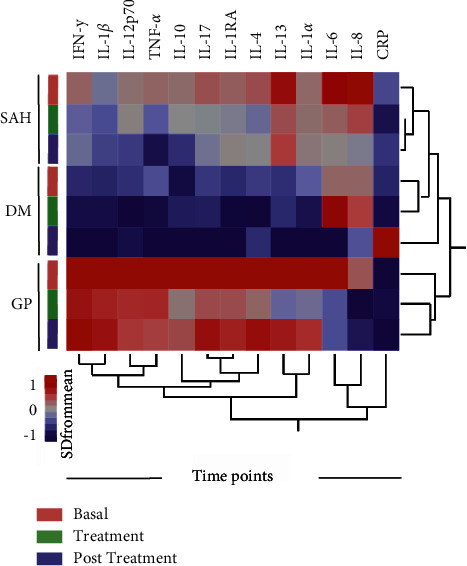
Heatmap demonstrating the inflammatory profile of patients according to the main aetiologies of kidney disease. The heatmap shows data for the mean plasma concentration of each indicated marker according to the main aetiologies of kidney disease, and the data were log-transformed and *Z* score normalized. A hierarchical cluster analysis (Ward's method with 100X bootstrap) was used to group the biomarkers with similar distributions between the time points. Dendrograms represent Euclidean distance. DM: diabetes mellitus; GP: glomerulopathies; SAH: systemic arterial hypertension.

Heatmap analysis identified a cluster of more inflamed individuals at baseline according to the aetiology of CKD. Patients with primary glomerulopathies (GPs) showed a pronounced inflammatory cytokine expression profile in plasma compared to patients with other aetiologies of CKD (diabetes or arterial hypertension) (Figure 4).

### 3.3. Secondary Outcomes

Treatment with EPP-AF at the dose studied was found to be safe in this population with no description of adverse reactions or the need to discontinue the treatment. The biochemical data for the evaluation of liver, pancreatic, and muscle enzymes are described in Table 4. There was no significant variation in these markers with EPP-AF® treatment.

## 4. Discussion

To the best of our knowledge, this is the first randomized study using propolis in patients under haemodialysis. In this study, we observed a high baseline inflammatory state in this population composed of patients on conventional haemodialysis and a significant reduction in some of these inflammatory molecules after the use of EPP-AF. Interestingly, by comparing the clinical groups, we observed that patients with glomerulopathies exhibited a unique biosignature characterized by a distinct profile of proinflammatory interleukins before treatment. There are no data in the literature comparing the degree of systemic inflammation in dialysis patients due to primary glomerulopathies with other aetiologies (diabetes mellitus or hypertensive nephrosclerosis), and further studies may give strength to our findings.

Proinflammatory cytokines are higher in haemodialysis patients than in healthy people [19], and this increase is multifactorial and caused by a greater production and reduction of their clearance with reduced renal function [2, 7]. Some cytokines and other proinflammatory mediators are considered medium molecules, being part of the “uraemic toxins,” representing approximately 20% of these compounds, and they are not significantly removed by conventional haemodialysis [20]. Recently, important advances have been achieved in the haemodialysis technology, especially in new capillaries with a greater capacity for medium molecule clearance [21]. Removing proinflammatory molecules is an important target, but it may not be enough; reducing or modulating their production in a safe way may be an essential complementary therapy, especially in patients who do not have access to haemodiafiltration or are not yet on dialysis [2]. Previous studies testing the clearance of beta-2 microglobulin in haemodialysis have shown a rebound of this molecule with a peak plasma level after dialysis, which can also occur with other medium molecules, such as interleukins [22, 23]. In the present study, the use of EPP-AF reduced proinflammatory cytokines, and there was an important tendency to maintain them at lower levels after a 4-week period without treatment, which indicated a lasting immunological effect.

In haemodialysis patients, inflammation is related to monocyte activation and immune dysregulation with the production of proinflammatory cytokines, resulting in acute or long-term adverse events [5]. Some inflammatory mediators, such as tumour necrosis factor-alpha (TNF-*α*) and IL-6, have been identified as the cause of the proinflammatory state as well as the vascular calcification process in haemodialysis patients [5, 24]. In haemodialysis patients, it was already suggested an increase in Th17 cells and decrease in Treg cells, which denotes a functional imbalance and a greater predisposition to the release of proinflammatory molecules among this population [25]. Despite its reduced use, it has also been demonstrated a significant increase in IL-8 in haemodialysis patients when compared with healthy patients in a control group, which may indicate a greater monocyte activation [26]. In the present study, there was a significant reduction in IL-6 in the posttreatment period and in TNF-*α* and other cytokines, such as INF- *γ*, IL-1Ra, IL17, IL-13, and IL-8, after the use of EPP-AF. Previous studies on macrophage cell cultures have also demonstrated that propolis inhibits the production of IL-1*β*, an important component of the inflammasome inflammatory pathway [12]. Our findings supported experimental evidence that indicates the immunomodulatory capacity of Brazilian green propolis extract through innate and adaptive immunity [27–29].

Cardiovascular mortality in haemodialysis patients is still high despite the advances over the last few decades [3]. It has been suggested that inflammation plays an important role in atherosclerosis and cardiovascular disease [2, 4, 21, 30]. Hs-CRP is an inflammatory marker that has also been shown to be a predictor of cardiovascular outcome [18]. Interestingly, analysis of hs-CRP showed that there was an increase in the number of patients in the lower cardiovascular risk group (hs-CRP <1) during the EPP-AF treatment compared to the baseline period, but this increase was not statistically significant. The short treatment period may explain the modification of the cytokine pattern without a significant change in hs-CRP. In the present study, the impact on cardiovascular outcomes was not measured by the short period of treatment, and long-term studies will be needed to clarify this hypothesis.

The use of convective clearance (HDF), unlike conventional HD, is associated with the removal of medium molecules with proinflammatory potential [31]. TNF-alpha (17 kDa) and IL-6 (26 kDa) are considered medium molecules, and they have clearance in continuous modalities of the renal replacement therapy using high cut-off capillaries, but the same does not occur in conventional haemodialysis [32]. A recent study in a paediatric population has shown that the use of haemodiafiltration attenuates the inflammation status more than conventional haemodialysis [33]. Another study has suggested the potential benefit in endothelial protection when using convective clearance [34]. However, HDF is still poorly available due to its high cost, and most haemodialysis patients use classical therapy, which does not effectively remove medium molecules. In the present study, EPP-AF reduced these proinflammatory markers independent of dialysis as all patients were on conventional haemodialysis.

The present study had several limitations. This was a single-centre study with a relatively small sample size, thus requiring additional studies in other and larger populations. Although the patients kept control of their use of medication, the study was open and there was no use of placebo. However, the present study demonstrated the safety of EPP-AF at a 200 mg/day in haemodialysis patients and assessed data on inflammation that bring information that can be used in the clinical practice.

Propolis has several properties of interest in medicine and nephrology, and some studies have suggested good prospects of propolis. Due to its variable composition and dependence on geographic region, flora, and climate, the use of standardized and validated extracts of propolis, such as EPP-AF®, is essential. An experimental study in a rat sepsis model has reported the renal protection capacity of EPP-AF® as an antioxidant and as an anti-inflammatory molecule due to its ability to reduce the expression of toll-like receptor 4 (TLR-4) and nuclear factor-kappa B (NF-kB) as well as cytokines in the renal tissue [11].

## 5. Conclusions

The present study demonstrated that in this population of CKD patients under conventional haemodialysis, there was a significant increase in proinflammatory cytokines, especially in patients with primary glomerulopathies. The Brazilian standardized green propolis extract (EPP-AF®) was safe and associated with a significant and lasting reduction in proinflammatory molecules. Randomized studies with a longer period of time and a larger cohort will be necessary to assess the impact of this modulation on CKD complications.

## Figures and Tables

**Table 1:** Baseline characteristics of patients (*n* = 37).

Characteristics	Results
Age, years, mean ± SD	58.6 ± 15
Male, *n* (%)	22 (59)
CKD aetiology, *n* (%)	
Diabetes	9 (24.3)
Hypertension	14 (37.8)
Glomerulopathy	6 (16.2)
Others	8 (21.6)
Haemodialysis access, *n* (%)	
Arteriovenous fistula	26 (70.3)
Tunnelled catheter	11 (29.7)
Dialysis vintage, months (mean, interval)	24 (4–228)
Heart failure, *n* (%)	10 (27)
Kt/V	1.4
Hb (g/dl)	11.3 (7.1–13.6)
Ferritin ng/mL	466 (32.2–1340)
Albumin g/dL	4.1 (2.9–4.5)
PTH pg/mL	201 (3.4–1794)

Values represent the median with the range (minimum and maximum values), whereas sex and causes of chronic kidney disease (CKD) were plotted as frequencies. Hb: haemoglobin; HD: haemodialysis; Kt/V: ratio for measuring dialysis treatment adequacy; PTH: parathormone.

**Table 2:** Inflammatory and immunological parameters.

Parameters	Time			*p* values	
	Baseline (*t*1)	Treatment (*t*2)	Posttreatment (*t*3)	*t*1 vs. *t*2	*t*1 vs. *t*3
IFN-y (pg/ml)	12 (11–22)	11 (10–16)	10 (8–46)	0.005	0.003
IL-10 (pg/ml)	27 (22–32)	27 (21–32)	25 (17–36)	0.20	0.03
IL-12p70 (pg/ml)	11 (10–15)	10 (8–14)	10 (8–21)	0.11	<0.001
IL-13 (pg/ml)	14 (8–52)	10 (6–26)	12 (5–40)	0.042	0.012
IL-17 (pg/ml)	15 (12–20)	12 (10–16)	13 (9–28)	0.039	0.003
IL-1Ra (pg/ml)	28 (18–47)	25 (15–38)	19 (14–55)	0.008	0.016
IL-1*α* (pg/ml)	19 (12–51)	19 (11–48)	20 (9–71)	0.060	0.033
IL-1*β* (pg/ml)	15 (10–44)	12 (9–30)	11 (8–30)	0.13	0.002
IL-4 (pg/ml)	18 (13–37)	14 (10–29)	17 (12–36)	0.12	0.40
IL-6 (pg/ml)	93 (52–164)	76 (42–170)	68 (33–137)	0.051	0.003
IL-8 (pg/ml)	556 (282–880)	323 (215–566)	358 (222–643)	0.009	<0.001
TNF-*α* (pg/ml)	155 (112–215)	120 (89–152)	109 (76–146)	<0.001	<0.001

IL: interleukin; IFN: interferon; TNF: tumour necrosis factor. *t*1 vs. *t*2: baseline vs. treatment; *t*1 vs. *t*3: baseline vs. posttreatment. Data are expressed as the median (IQR). *p* values were measured by the Wilcoxon signed-rank exact test and Wilcoxon signed-rank test with continuity correction.

**Table 3:** Hs-CRP values per stratum.

Characteristics	Baseline, *N* = 37^a^	Treatment, *N* = 37^a^	Posttreatment, *N* = 37^a^	*p* value Basal vs. treatment^b^	*p* value Basal vs. posttreatment^b^
Hs CRP (mg/L)					
<1	4 (11%)	8 (22%)	8 (22%)	0.3	0.3
1–3	14 (38%)	12 (32%)	15 (41%)		
>3	19 (51%)	17 (46%)	14 (38%)		

Hs-CRP: high-sensitivityC-reactive protein. ^a^*n* (%). ^b^McNemar's chi-squared test.

**Table 4:** Biochemical safety data before and after propolis treatment.

Parameters	Baseline (*t*1)	Treatment (*t*2)	*p* value
Amylase (U/dL)	197 (156–252)	185 (139–242)	0.09
ALT (U/L)	15 (12–21)	14 (11–22)	0.62
AST (U/L)	19 (6–36)	18 (15–26)	0.74
CK (U/L)	64 (42–104)	79 (62–103)	0.81

ALT: alanine aminotransferase; AST: aspartate aminotransferase; CK: creatine kinase. Values represent the median (IQR). Differences between the pre- and posttreatment groups were examined using the Wilcoxon matched paired signed-rank test, represented in the “*p* value” column.

## Data Availability

The datasets generated during and/or analysed during the current study are available from the corresponding author on reasonable request.

## Abbreviations

ALT: Alanine aminotransferase
AST: Aspartate aminotransferase
CKD: Chronic kidney disease
CPK: Creatine phosphokinase
CV: Cardiovascular
HD: Haemodialysis
HDF: Haemodiafiltration
HsCRP: High-sensitivity c-reactive protein
IFN-*γ*: Interferon gamma
IL: Interleukin
NF-kB: Nuclear factor-kappa B
TNF-*α*: Tumour necrosis factor-alpha
TLR-4: Toll-like receptor 4.
